# Lymphatic Tissue Engineering and Regeneration

**DOI:** 10.1186/s13036-018-0122-7

**Published:** 2018-12-17

**Authors:** Laura Alderfer, Alicia Wei, Donny Hanjaya-Putra

**Affiliations:** 1 Department of Aerospace and Mechanical Engineering, Bioengineering Graduate Program, University of Notre Dame, Notre Dame, IN 46556 USA; 2 Department of Chemical and Biomolecular Engineering, University of Notre Dame, Notre Dame, IN 46656 USA; 3 Harper Cancer Research Institute, University of Notre Dame, Notre Dame, IN 46556 USA; 4 Center for Stem Cells and Regenerative Medicine, University of Notre Dame, Notre Dame, IN 46556 USA; 5 Advanced Diagnostics and Therapeutics, University of Notre Dame, Notre Dame, IN 46556 USA; 6 Center for Nanoscience and Technology (NDnano), University of Notre Dame, Notre Dame, IN 46556 USA

**Keywords:** Lymphangiogenesis, Tissue Engineering, Disease Modeling, Wound Healing, Lymphedema, Stem Cells, Biomaterials, Interstitial Fluid, Regeneration

## Abstract

The lymphatic system is a major circulatory system within the body, responsible for the transport of interstitial fluid, waste products, immune cells, and proteins. Compared to other physiological systems, the molecular mechanisms and underlying disease pathology largely remain to be understood which has hindered advancements in therapeutic options for lymphatic disorders. Dysfunction of the lymphatic system is associated with a wide range of disease phenotypes and has also been speculated as a route to rescue healthy phenotypes in areas including cardiovascular disease, metabolic syndrome, and neurological conditions. This review will discuss lymphatic system functions and structure, cell sources for regenerating lymphatic vessels, current approaches for engineering lymphatic vessels, and specific therapeutic areas that would benefit from advances in lymphatic tissue engineering and regeneration.

## I. Introduction to the Lymphatic System and its role

### Function

The lymphatic system is nearly ubiquitous in the human body, present in all tissues except the epidermis, cartilage, eye lens, cornea, retina, and bone marrow [1, 2]. The main functions of the lymphatic system include fluid homeostasis and interstitial fluid drainage, immune cell surveillance and trafficking, and lipid absorption [1, 3–6]. Lymphangiogenesis, the process of forming new lymphatic vessels from pre-existing vessels, not only occurs during development but also in adults during wound healing, inflammatory responses, and the cancer microenvironment [1, 7].

The lymphatic system includes bone marrow and the thymus, classified as central or primary lymphoid organs, as well as lymphatic vessels, lymph nodes, spleen, adenoids, Peyer’s patches, appendix, and lymphoid tissue, classified as peripheral or secondary lymphoid organs [8]. Within the cellular microenvironment in tissues, the fluid, proteins, solutes, and extracellular matrix (ECM) are collectively termed the interstitium [4]. Interstitial fluid (IF) is a plasma filtrate that is generated by transcapillary filtration and is governed by Starling forces, the net difference between hydrostatic and osmotic pressures, at the microcirculatory level [9]. In order to maintain fluid homeostasis, lymph formation in the initial lymphatic vessels must be balanced by the net flux of plasma being filtered out [4]. Transport of IF from the initial capillaries to the collecting vessels is facilitated by IF pressure and systemic forces, including blood pressure, respiratory motion massage, peristaltic movement, and contractility of surrounding skeletal muscle [10–14]. As a result of constantly clearing IF, the lymphatic system is chronically exposed to and stimulated by fluid flow and pressure [5].

IF is transported via lymph vessels to lymph nodes and then returned back to the blood circulation. Properties of the lymphatic capillary wall, hydrostatic pressure, and protein concentrations in the blood and interstitium are determining factors in the formation of IF [4]. Contained within IF are macromolecules, dissolved solutes, viruses, bacteria, certain leukocytes, and cell debris [1]. IF facilitates the transportation of various molecules between local sites and tissues, including nutrients, waste products, signaling molecules, antigens, and cytokines. The specific composition of IF depends on pathogenesis, inflammatory responses, and the nearby organs or tissues [4]. Under healthy conditions, IF will comprise approximately 20% of the body’s weight and 2-4 liters of IF will be returned to the venous vasculature from the lymphatic system daily [1, 15]. IF volume is constantly maintained by interstitial buffering mechanisms [8], which include structural alterations, differences in forces acting across the capillary wall, and lymph flow [4].

### Structure

Despite the lymphatic system being so extensive, the field of lymphatic research is very young due to lymphatic specific markers being discovered only 20 years ago. Since the identification of lymphatic specific markers and isolation of lymphatic endothelial cells, key differences between the vascular and lymphatic systems have been identified, allowing for specific research efforts into the lymphatic system without results being confounded by the inclusion of the vascular system [4].

Several key differences exist between blood vessels and lymphatic vessels. Composed of blood endothelial cells (BECs), blood vessels exhibit tight junctions and a continuous basal lamina. Conversely, lymphatic vessel (LVs), composed of a single layer of lymphatic endothelial cells (LECs), have a discontinuous basal lamina as a result of overlapping and interdigitated endothelial cells [4, 16], blind ended sacs [16], and a wide lumen [2]. Additionally, lymphatic capillaries lack pericytes, smooth muscle cells (SMCs), and mural cell coverage [3, 17]. The ECM and lymphatic capillaries are connected with anchoring filaments and when the interstitial volume increases, these anchoring filaments are pulled apart which causes lymphatic valves to open [18, 19]. These anchoring filaments are composed of collagen VII [20, 21], transmembrane integrins, and focal adhesion kinase [17]. VE-cadherin joins discontinuous and overlapping endothelial cells together into buttonlike patterns [22, 23] which are postulated to serve as one-way flaps that facilitate the absorption of cells, fluid, and proteins [4]. IF enters LVs through these button-like junctions and is facilitated by the pressure gradient [22].

Unlike the circulatory system, the lymphatic system is a one-way drainage system that originates in tissues and organs, is funneled through a series of many small vessels emptying into fewer larger vessels, and empties into the circulatory system [5]. Continuous fluid flow between blood capillaries and tissues is achieved by lymphatic capillaries absorbing excessive fluids from the interstitial space which simultaneously provides nutrients to cells, eliminates waste products, and dissipates interstitial pressure buildup [24]. In the larger collecting lymphatics, valves assist in lymph propulsion and also prevent retrograde flow, ensuring a unidirectional propulsion of lymphatic fluids [4]. Muscle contractions by the surrounding tissues as well as blood pressure also assist in creating this unidirectional propulsion [14, 25].

In addition to these general characteristics of the lymphatic system that can be found throughout the body, there are also several specialized functions or notable lymphatic features within organ systems. In the case of regulating lipid uptake in the gastric lymphatic system, lacteals, specialized lymphatic vessels, are positioned in the villi of lumen next to blood capillaries [26]. Endothelial cells, along with keratinocytes, fibroblasts, macrophages, and platelets are involved in the wound healing process [27]. In the case of inflammation, the gene expression of LECs is altered and leads to the lymphatic network expanding, along with increased fluid drainage both to and from the site of inflammation [28]. LVs also contribute to the inflammatory response by draining cytokines and chemokines [26]. The heart contains an extensive lymphatic network, and combined with the role of the lymphatic system in inflammation, targeting lymphangiogenesis in the heart after myocardial infarctions to improve recovery has become an area of interest [29, 30].

## II. Complications Associated with the Lymphatic System

**Fig. 1:**
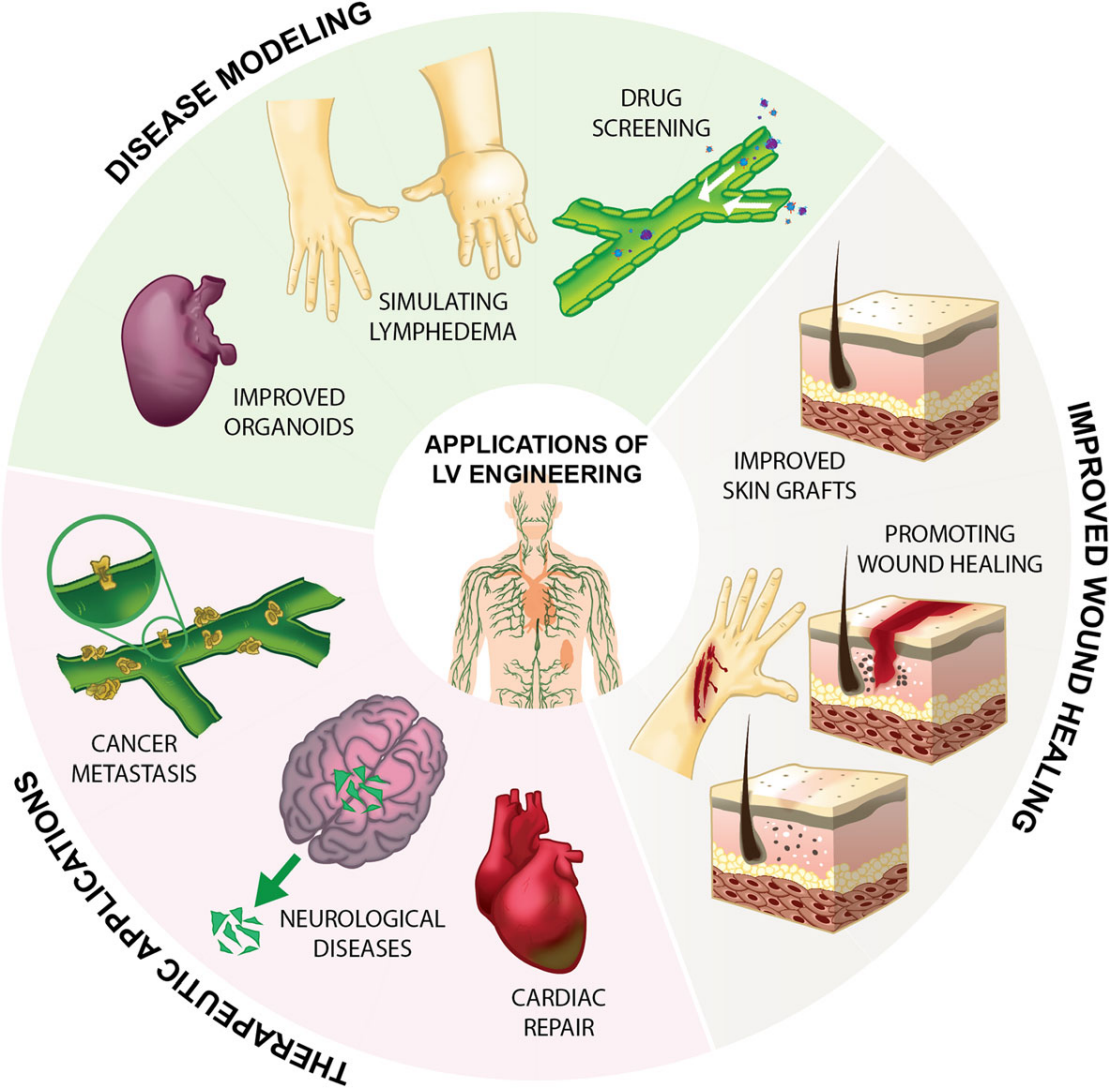
Multiple areas of medicine and disease pathologies could benefit from advances in lymphatic tissue engineering. These include rescuing cardiac tissue after MI, clearing macromolecules from the brain to slow or hinder the onset of Alzheimer's disease, further understanding the pathways of cancer metastasis in order to effectively target cancer progression, designing improved organoids which would more accurately model native tissue, simulating lymphedema as an experimental model that could be used to design treatments for lymphedema beyond mechanical pumping, screening potential therapeutic agents to understand how they impact and interact with the lymphatic system, engineering superior skin grafts that incorporate the dermis and associated functionality, and promoting wound healing

Complications associated with the lymphatic system span a wide spectrum, including congenital disorders, cancer and side-effects of cancer treatments, cardiovascular disease, diabetes, and parasitic infections [25, 31]. While some lymphatic disorders are genetically related, lymphatic complications most often arise as a secondary complication following cancer, cardiovascular disease, and immunological diseases [32]. Specific pathologies and areas that could benefit from improved lymphatic function or engineered lymphatic tissue are summarized in Fig. 1.

### Lymphedema

Lymphedema, characterized by chronic swelling of an extremity, results from local accumulation of interstitial fluid due to insufficient lymph drainage [4] and is one of the most prevalent lymphatic-dysfunction conditions [24]. Globally, up to 250 million people are affected by lymphedema with the most prevalent cause being the parasitic disease filariasis [33]. In developed countries, the most common cause of lymphedema is disruption of lymphatic pathways, typically from cancer treatments in the form of tumor removal or radiation. The swelling of soft tissues from lymphedema results in discomfort [24], lack of mobility, and other health complications, both disfiguring and disabling a patient due to excessive swelling, reduced mobility, and social stigma associated with the condition. A patient’s quality of life is significantly reduced on a physical, mental, social, and economic basis [34]. Beyond reducing the affected person’s quality of life, lymphedema also leads to complications in the immune response [31].

There are two classes of lymphedema; primary and secondary. Primary lymphedema results from genetic disorders and occurs in 1.15/100,000 people [35]. Tissue trauma, surgical removal of a tissue and the associated lymphatic tissue, or radiation therapy-related damage in non-obese patients are the major causes of secondary lymphedema [35]. The lymphatic endothelium is ruptured after a wound and compromises the draining capacity of LVs, resulting in lymphedema [36–38]. Chronic lymphedema affects 0.13-2% of the global population [39]. In the case of breast cancer patients following a mastectomy, 24-49% of patients develop upper extremity lymphedema [40].

There are multiple causes of lymphedema. Dysfunction of lymphatic fluid uptake [5], disruptions to the lymphatic system due to injury, disease, or surgery [41], congenital absence, radiation therapy, infection, and trauma can result in lymphedema [42]. Lymphedema commonly occurs in patients that undergo lymph node resection for cancer treatment [43] and the extent of axillary surgery influences lymphedema development [42]. These patients experience progressive and chronic swelling, recurrent infections, pain, and a significantly decreased quality of life [44, 45].

### Cancer Progression and Metastasis

Lymphangiogenesis, as well as immune suppression and tolerance, have been positively correlated with cancer progression [9]. In the tumor microenvironment and tumor-draining lymph nodes, lymphangiogenesis is more specifically correlated with invasion, metastasis, and poor prognosis [1, 46, 47]. Most carcinomas initially metastasize to the lymph nodes [9], and from there can metastasize through the body using the lymphatic system as a circulation route. Tumors frequently recruit the lymphatic system as a means to metastasize. Additionally, the matrix stiffens and the immune microenvironment of a tumor is altered by stromal cells as a mechanically stress-induced response to the increased lymph flow [9].

### Cardiovascular Disease

In many cardiovascular diseases, including myocardial infarction (MI) and chronic heart failure, myocardial edema occurs. A growingly accepted hypothesis is that insufficient cardiac lymphatic transport is associated with cardiovascular pathologies [2, 48, 49]. Following a MI, there is an endogenous cardiac lymphangiogenic response [29]. Despite this response, chronic myocardial edema and inflammation-aggravating cardiac fibrosis and dysfunction persists due to the remodeling and dysfunction of lymphatic collecting ducts [29].

### Impaired Wound Healing

If the removal of local debris and inflammatory cells is delayed, or local interstitial fluid is chronically present, the wound healing process is impeded [50–52]. A reduction in P_IF_, the interstitial fluid pressure in an interstitial compartment, during tissue injury has been identified as a major factor in the development of acute edema [4]. In the case of chronic inflammation, lymphangiogenesis is upregulated and a higher LV density can be observed in these areas [7, 53–56]. In a mouse study, it was observed that inflammatory lymphangiogenesis could aid in clearing edema fluid and antigens, thereby promoting the wound healing process if lymphangiogenesis is upregulated [4, 57].

### Obesity

Mice studies have revealed that a high-fat diet led to lymphatic vessel dilation and decreased diffusion capacity of lymphatic capillaries, resulting in impaired lymphatic transport and vessel function [58, 59]. In obese patients, defined by a body mass index (BMI) greater than 40, benign hyperproliferative lymph tissue was a hallmark of massive localized lymphedema [60]. While it is not yet clear if obesity directly causes lymphatic abnormalities, there is a correlation. Additionally, cardiopulmonary and renal disease is related to obese patients who experience an aggravation of edema [61].

## III. The Origin of Lymphatic Vasculatures

**Fig. 2:**
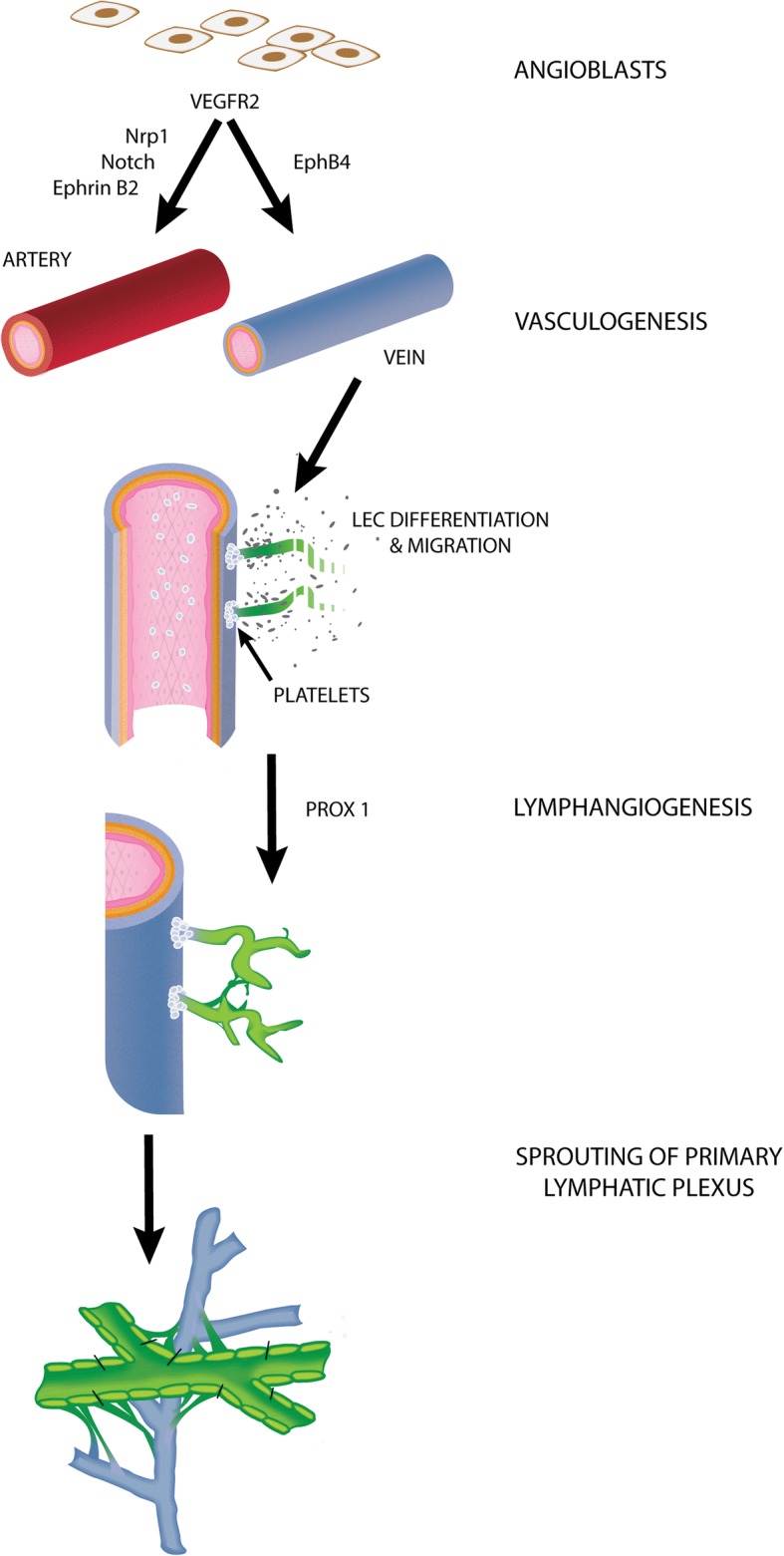
During vasculogenesis angioblasts assemble into primitive capillary plexus, which can further differentiate into either arteries through Ephrin B4 signaling or veins through Neuropilin, Notch, and Ephrin B2 signaling. Platelet aggregation in cardinal vein allows lymphangiogenesis to occur. A gradient of signaling molecules such as VEGF-C, signals the for the for LEC differentiation and migration, forming the primary lymphatic plexus. The lymphatic plexus begins to sprout and mature into lymphatic vessels

The assembly of angioblasts to form *de novo* blood vessels is known as vasculogenesis [62]. During early stages of the embryo, the dorsal aorta and cardinal vein are formed by vasculogenesis [63], where vascular endothelial growth factor receptor 2 (VEGFR-2) plays an important role [64, 65]. Vasculogenesis begins when signals from the visceral endoderm affect the fate of mesoderm precursors to endothelial cell lineage [66, 67]. Lymphangiogensis is the centrifugal development of LECs from the venous endothelial cells of cardinal veins, forming a vascular network that is distinct from the arteries and veins within the system (Fig. 2) [2, 68–70]. For vessel separation to occur, the inhibition of proliferation and migration of LECs by activated platelets is necessary [71, 72]. Throughout vertebrate development, the vascular network has to constantly remodel and adapt to the changes in neighboring tissues [73]. Within mouse embryonic models, primary lymphatic sacs have been found to be derived of endothelial cell clusters from the cardinal veins that have committed to the lymphatic phenotype [2, 74]. Centrifugal growth then allows the lymphatic system to continue developing [72]. Disruption of normal blood and lymphatic vessel development often leads to disease phenotypes or embryonic lethality [73, 75, 76].

Furthermore, the function of the lymphatic system is to drain the interstitial fluid from neighboring tissues [2, 77]. This implicates lymphatic system separation from the blood and venous circulation is critical during development [2, 78]. This process has been shown to be mediated by O-glycosylation of podoplanin (PDPN) on LECs due to its interaction with platelets and lectins during development to maintain stable platelet adhesion and aggregation under sheer stress [2, 72, 79, 80]. PDPN is a lymphatic marker that is expressed by the LECs of cardinal veins and not by blood vascular endothelial cells [81–83]. Besides expression in the lymphatic endothelium, PDPN is also expressed by peritoneal mesothelial cells, osteocytes, glandular myoepithelial cells, ependymal cells, stromal reticular cells, and follicular dendritic cells in lymphoid organs [81]. Lymphatic endothelium O-glycans have been shown to play a role in maintaining the distinct blood and lymphatic systems by protecting and maintaining the proper function of endothelial PDPN [72, 79]. In experiments where there was an O-glycan deficiency, PDPN expression was downregulated, causing the non-distinct blood and lymphatic systems [75]. Mice lacking PDPN were unable to survive past birth due to respiratory defects resulting from the inability of the lymphatic sacs to grow from the cardinal veins [84]. Lymphatic vasculature also failed to develop in mouse embryonic models with prospero homeobox protein (PROX1) knockouts [85]. C-type lectin-like receptor 2 (CLEC-2) is a platelet activation receptor for PDPN that has roles in cancer and lymphangiogenesis and is expressed in other blood cell types [82, 86].

The lymphatic system is also involved in the immune defense of vertebrates and has been shown to be involved in the progression of cancer and other diseases [2, 77]. Lymph nodes allow lymphocytes to circulate as part of the immune defense system [87, 88]. The lymphatic system also functions as a highway for cancer metastasis [85]. Lymph-node involvement also plays an important role in tumor metastasis [89, 90]. Vascular endothelial growth factor C (VEGF-C) and vascular endothelial growth factor D (VEGF-D) can also increase the vascular permeability of tumor cells and change the adhesive properties of the lymphatic endothelium [2, 89].

## IV. Vascular Beds

The three vascular beds, arterial, venous, and lymphatic system, form the circulatory system [91].

Since various research disciplines within vascular biology are focusing more and more on the use of organotypic and vascular bed-specific cell origins, here we will review different LECs derived from different vascular beds (e.g., intestinal crypt, lymph node), eye (Schlemm’s canal), and brain (Glymphatics).

### Intestinal Crypt

**Fig. 3:**
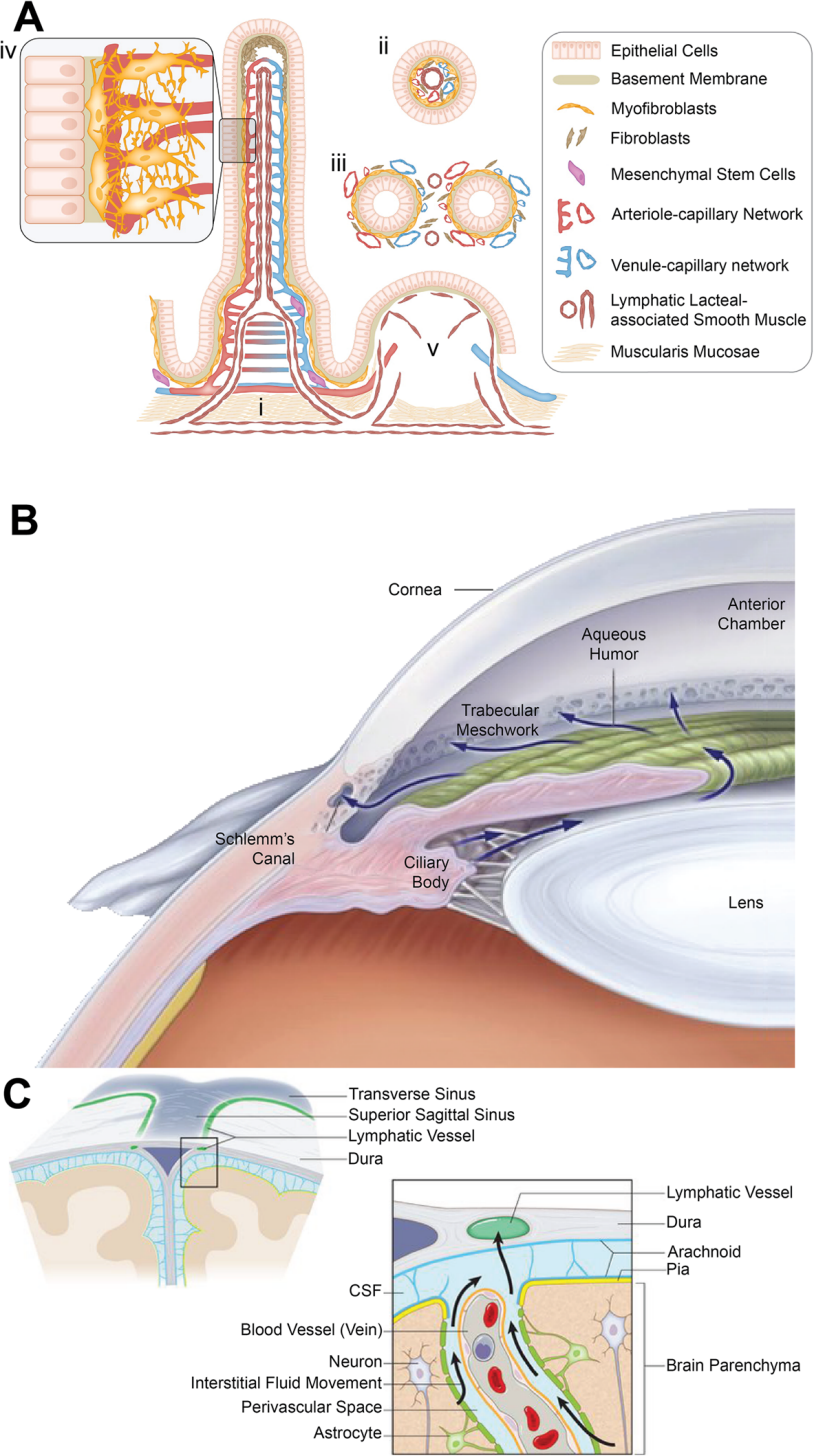
A schematic representation of different vascular beds. (**a**) Intestinal crypt. [i] A longitudinal dissection showing the anatomy of the villus and intestinal crypt. [ii] A cross-sectional view of the villus. [iii] A cross-sectional view of the intestinal crypt. [iv] An increased magnification to a portion of the villus to show the interactions between myofibroblasts and pericytes with the basement membrane and neighboring capillary network. [v] This depicts a Peyer's patch. Illustration in panel A was adapted with permission from [269]. (**b**) Schlemm's Canal. The Schlemm's canal is responsible for draining the aqueous humor from the trebecular meshwork to the spiscleral venous system. Although to a lesser extent, ciliary bodies are also involved in draining the aqueous humor. Illustration in panel B was adapted with permission from [100]. (**c**) Glymphatics. Interstitial fluid and CSF drain from the CNS and surrounding tissues through the glymphatic system. Illustration in panel C was adapted with permission from [109]

Within the intestine, there are mucosal glands known as crypts. The epithelium of the intestinal tract is constantly renewed through the highly proliferative epithelial cells housed within these crypts [92]. When these intestinal epithelial cells undergo apoptosis, they are endocytosed by a subset of dendritic cells and transported to T cell areas of the mesenteric nodes [93]. Furthermore, lymphatic vessels in the colon occasionally branch through the muscularis mucosae to reach the basal colonic crypts (Fig. 3a) [94]. Increased lymphatic vessels in both the lamina propria and submucosa of the intestine has been correlated with chronic inflammatory bowel diseases [94]. Further study of the stem cell origin and potentially lymphatic origin within the intestinal crypt and their roles in disease states are needed.

### Lymph Node

As previously mentioned, the lymphatic system is involved with immune defense. More specifically, LECs interact with the immune cells within the lymph nodes [95]. It has been shown that lymph node LECs contain molecules, such as human leukocyte antigen, that are needed for T cell activation in the immune system [95]. However, these LECs may also play an inhibitory role in dendritic cell-induced allogenic T cell proliferation [95]. The involvement of lymph node LECs with the immune system goes beyond its involvement with T cells. They also express multiple antigens on their peripheral tissues that are independent from the automimmune regulator, suggesting their role as mediators of peripheral immune tolerance [95].

### Schlemm’s Canal (Eye)

Schlemm’s canal is an endothelium-lined vessel that encloses the cornea [96] and separates the aqueous humor from systemic circulation [97]. Previously, it was unknown whether Schlemm’s canal functions as a blood or lymphatic vessel. Through studies utilizing lymphatic specific markers and gene expression of PROX1, Schlemm’s canal was found to have a lymphatic phenotype (Fig. 3b) [96, 98]. However, other studies have shown Schlemm’s canal endothelia to have characteristics to both blood capillary and LECs, along with some unique characteristic of its own [97, 99]. Dysfunction of Schlemm’s canal can lead to disease states such as glaucoma, a condition where degradation of the optic peripheral nerves ,. leads to loss of vision [100], and patients with glaucoma have been found to have smaller Schlemm’s canal [101].

### Glymphatics (Brain)

As part of our nervous system, the brain and spinal cord form the central nervous system (CNS). Surrounding the brain and spinal cord is a clear and colorless body fluid known as the cerebrospinal fluid (CSF). Historically, it was assumed that the CNS did not have any lymphatic vasculature [102, 103]. In recent studies, the glymphatic system, a glial-dependent perivascular network with a lymphatic function has been discovered within the brain [103, 104]. Together the CSF and the interstitial fluid of surrounding tissues drain from the CNS to regional lymph nodes (Fig. 3c) [105]. More importantly, CSF fluid drains through lymphatic vessels and thus has important interactions with the immune system such as antigen-presenting cells [106–109]. In contrast, the interstitial fluid in the CNS drains through the walls of cerebral capillaries and arteries, which do not allow the transport of antigen-presenting cells [110, 111]. The involvement of the lymphatic system in fluid flow through the CNS has been shown to be involved in Alzheimer’s disease [112, 113] and multiple sclerosis [114]. Here, it is important to note that the CNS anatomy itself does not have defined lymphoid tissuess [115].

## V. Differences between BECs and LECs

Increased expression of versican, collagens, laminin, N-cadherin, and many other ECM components, along with adhesion molecules specific to the blood vascular endothelial cells have been identified [116]. Historically, it has been difficult to identify lymphatic vessels due to a lack of lymphatic specific markers. Distinct molecular markers for lymphatic vessels such as PDPN, VEGFR-3, PROX1, and lymphatic vessel hyaluronan receptor-1 (LYVE-1) have since been identified [2]. It should be noted that within a vertebrate, imaging the lymphatic system using magnetic resonance lymphangiography by utilizing injected contrast media is possible [117]. Other imaging methods involve lymphoscintigraphy, fluorescence microlymphangiography, and NIR fluorescence lymphatic imaging [117, 118].

There are some theories on how the lymphatic system forms. Notably, Sabin predicted that primary lymphatic sacs are derived from endothelial cells that bud from veins and form the capillaries around tissue and organs through centrifugal development [119, 120]. This theory is supported by the venous endothelial cells expressing PROX1 [85] as well as various genetic studies in zebrafish models [121, 122]. The lymphatic system can be a low flow, low pressure system because of its specialized anchoring filaments that allow the lymphatic vessels to stay open despite increased tissue pressure [2]. Furthermore, lymphatics have significantly less platelets and erythrocytes and thus are less coagulable [2, 123].

Due to some of their similarities, the lymphatic vessels may have a shared origin with blood vessels [2]. This may explain some of the similarities between lymphatic and blood vessels. Both are lined by endothelium, surrounded by SMCs, and are stimulated by some common growth factors [2, 90]. Notably, PROX1 is overexpressed ectopically in blood endothelial cells, about one third of LEC specific gene expression [116, 124, 125]. The lymphatic vessels of mammals are lined by endothelial cells that may have developed from embryonic veins due to their dependence on PROX1 and VEGF-C signals [69, 83, 85, 126, 127]. VEGF-C is necessary for endothelial cells expressing PROX1 to migrate and form lymph sacs [127]. Besides VEGF-C, VEGF-D also induces the development of LECs [77]. Both VEGF-C and VEGF-D bind to endothelial cell specific tyrosine kinase receptors VEGFR-2 and VEGFR-3 [77]. VEGFR-2 is crucial in angiogenesis, the formation of new blood vessels from pre-existing blood vessels, and VEGFR-3 on LECs is responsible for lymphangiogenesis, the growth of lymphatic vessels [126, 127]. Interestingly, the gene product expression for VEGFR-3 only develops as the embryonic growth progresses [85, 123]. This suggests that the lymphatic system develops in a step process following other signals yet to be identified.

Zebrafish embryos develop lymphatic vessels as a function of VEGF-C and the receptor VEGFR-3 signaling [69]. This result was also discovered in mice models [72]. Similarly, the expression of angiopoietin 2 (ANG2) also affects the development and function of the lymphatic system for both mice and zebrafish models [69, 128]. It is important to note that although ANG2 has a role in lymphatic differentiation and maturation, it does not have a role in the sprouting and segregation of lymphatic sacs [72]. The lymphatic system also plays a role in zebrafish meningeal vascularization through the meningeal mural lymphatic endothelial cells (muLECs) that surround these meningeal blood vessels and ensure their normal development [68]. muLECs may have roles in angiogenesis and vessel maintenance due to its expression of LEC marker genes and vascular endothelial growth factors [68]. As previously mentioned, either primary or secondary lymphedema can result in the dysfunction of the lymphatic system [129]. Primary lymphedema is inherited, while secondary lymphedema is acquired [129]. Current methods have been unable to treat lymphedema. A few promising methods to treat lymphedema are to use mesenchymal stem cells, adipose-derived regenerative cells, and other cell-based therapies [30, 130]. Benefits of utilizing adipose tissue involve its low risk and high yield along with the numerous cell types present such as adipocytes, vascular endothelial cells, and vascular SMCs [131]. More importantly, some of the cells present in adipose tissue can differentiate into cardiac muscle, endothelium, cartilage, and many other lineages [131]. Future studies should address the role of lymphatic system in lymphedemic diseases.

PDPN is expressed in LECs, but not in vascular endothelial cells [82]. As such, vascular endothelial cells cannot interact with CLEC-2 [82]. Similar to mice lacking PDPN, mice with deficiency in CLEC-2 had incomplete separation between the blood and lymphatic system [82, 132]. Bone morphologic protein-9 (BMP-9) is activated by the CLEC-2 and PDPN interaction [82, 86]. BMP-9 may be responsible for the role platelets have in regulating the separation of the lymphatic vessel from the blood and venous circulation through the inhibition of LEC proliferation, migration, and tube formation [82]. Hyaluronan (HA) is a large glycosaminoglycan that is crucial for cell migration and morphogenesis during development [133–136]. The first homologue of the CD44 HA receptor detected was the lymphatic vessel hyaluronan receptor-1 (LYVE-1) [77, 137]. More importantly although CD44 is expressed in some progenitor endothelial cells [138, 139], LYVE-1 is predominantly expressed on lymphatic vessels and not on blood vessels [137]. Consequently, LYVE-1 has been shown to be the first marker for lymphatic endothelial commitment [77, 137]. In adults, LYVE-1 expression remains high in the lymphatic capillaries, but becomes downregulated within the collecting lymphatic vessels [77]. In summary, PROX1, VEGFR-3, PDPN, and LYVE-1 are all LEC specific markers.

## VI. Demand for Engineered Functional Lymphatic Vessels

The demand for engineered, functional lymphatic vessels can be divided into two main categories; therapeutic solutions and model systems for future scientific discoveries. Currently, the only therapeutic options for patients with lymphatic dysfunction include mechanical or manual lymph drainage, compression garments, or microsurgery [44, 45]. While these treatments reduce the edema volume, they are only transient solutions and require patients to use them for a lifetime. Chronic treatments, combined with superficial and transient improvements, places a large burden on the healthcare system and patients [140]. When taking into account a rising life expectancy and an increasingly sedentary lifestyle, the number of people affected by complications of the lymphatic system is going to increase in the future [24].

### Therapeutic and Clinical Solutions

Surgical procedures aim to limit fluid accumulation, but when these attempts are unsuccessful, patients are limited to supportive care as their only remaining option. Surgical approaches are complex and include lymphatic bypass surgery and lymph node transfer [42, 141]. While the long-term outcome of these procedures is better than nonsurgical interventions, only early stage lymphedema patients are candidates [24]. In the case of early stage lymphedema in the upper limb region, 15-60% of patients have no improvement in limb volume after surgery [142]. In the case of advanced lymphedema, surgical treatments are completely absent [143].

Therapeutic applications of engineered lymphatic vessels include treating edema, aiding or improving the wound healing process, creating superior skin grafts, vascularizing engineered organs in order to make them viable transplantation solutions, and offering tissue replacement options for post-tumor removal. Engineered lymphatic vessels, including lymphatic organs such as the spleen, can be transplanted to improve or repair deficiencies that originated from disease or injury [50]. Depending on the severity of the lymphatic related disease, replacement of the dysfunctional lymphatic tissue may be required instead of repairing the existing tissue. While current surgical techniques include lymphatic bypass surgery or microsurgical LV transplantation, creating anastomoses is very difficult due to the thin and fragile walls of LVs [144, 145]. Functional skin grafts are essential for burn healing and plastic surgery, and the next critical step is the incorporation of vascular plexuses in autologous skin grafts [50–52, 138].

### Disease Modeling and Drug Screening

Excluding the lymphatic system, almost every major organ including the heart, lungs, liver, kidneys, nervous system, bone, and cartilage have been targeted with tissue engineering efforts to develop functional replacement tissues [146–152]. However, without blood and lymphatic vessels, these engineered replacements will not be fully viable solutions for in vivo applications [148, 153–155]. While in vitro blood vessel engineering gained interest over the past few decades due to the need to supply engineered tissues with nutrients [138, 156–159], lymphatic vessel engineering has lagged behind [41]. In vitro vascularization is a major barrier to and requirement for effectively transplanting engineered tissues and organs [160], highlighting the need for LV engineering in order to advance the entire field of tissue engineering.

Engineered lymphatic organs, including LVs, lymph nodes, and spleens, provide ex vivo research models [50]. A three-dimensional tissue construct with functional lymphatic vessels would allow for drug screening as well as a tunable disease model for in vitro experiments [161]. Additionally, a functional lymphatic model could be systematically probed to elucidate poorly known pathways, including diabetes and cancer metastasis [162–165]. It is known that the VEGF-C/VEGFR-3 signaling axis spurs the growth of LVs, but how this signaling axis is regulated in diabetes is poorly understood [166]. Bone-marrow mesenchymal stem cells (BM-MSCs) contribute to the progression of cancer by promoting angiogenesis, but their involvement in lymphangiogenesis is poorly understood [167]. Additionally, the effect of inflammatory lymphangiogenesis on immunity is not yet understood [9]. Cardiac lymphatic vessels are acknowledged, but their role in development as well as in diseased and healthy adult hearts remains virtually unknown [29, 48]. With a lymphangiogenesis model, the wound healing process could continue to be studied. Lymphedema may alter the composition of interstitial fluid, and analysis in a controlled model environment could advance the understanding on the pathomechanisms of lymphedema [4].

## VII. Stem Cells as Cell Source for Lymphatic Regeneration

Previous research has shown that functional vascular endothelial cells derived from hematopoietic stem cells from the adult mouse bone marrow were possible [168–171]. Molecules that are involved in hematopoietic cell differentiation have been found to be associated with various types of cancer [172]. Furthermore, these hematopoietic stem cells have also been found in both vascular and diseased vascular endothelia [168, 169]. Thus, the question of whether hematopoietic stem cells are involved in maintaining the normal function of the LEC remains to be answered. In a similar study, LECs derived from hematopoietic stem cells have been shown to successfully integrate itself into the lymphatic vessels for both normal and tumorigenic tissues [173]. This study also showed that acutely radiated circulating cells intervened between the hematopoietic stem cells and its involvement in the lymphatic endothelia [173]. The results of this study suggest that hematopoietic cells may be involved in maintaining lymphatic homeostasis and modification of these cells may aid in targeting diseases of the lymphatic system such as lymphangiomas or lymphangiectasias.

The precursors of LECs are less studied and known. Recent evidence indicate the process to differentiate embryonic stem cells to either hematopoietic cells or endothelial cells *in vitro* follow nearly identical pathways as within embryos [172]. Isolated progenitor cells from differentiating embryos and embryonic stem cells *in vitro* were able to elucidate these intermediate stages [174]. A recent study showed it was possible to differentiate VEGF-R2^+^ cells derived from embryonic stem cells into LECs by following LEC specific markers [172]. Multipotent adult progenitor cells (MAPCs) were shown to increase both capillary and pre-collector vessel regeneration in wounds [57]. Human MAPCs have also been found to be involved in the survival and reconnection of transplanted lymph nodes that allowed an increase in the functional role they had in the lymphatic vessels [57].

**Fig. 4.**
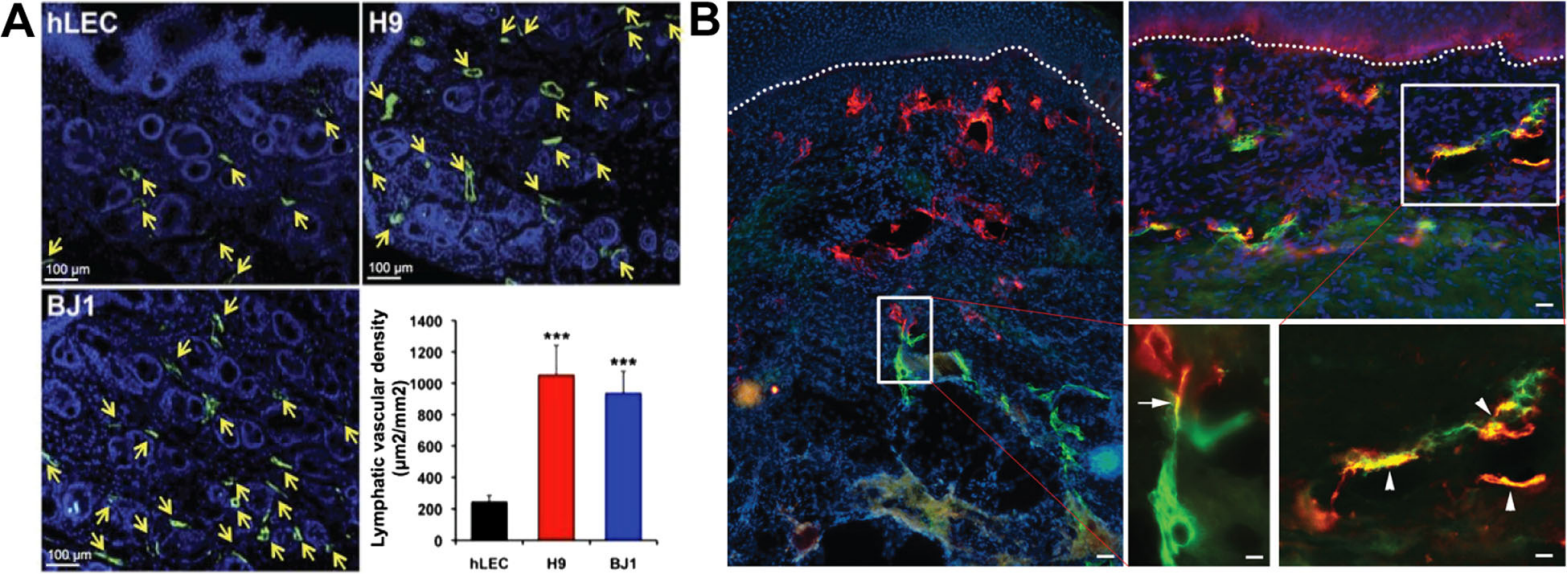
(**a**) LEC (LYVE-1^+^/Podoplanin^+^) cells derived from hPSCs (H9 and BJ1) were injected into the skin wound on the backs of nude mice. Lymphatic vessels indicated by arrows (LYVE-1) were significantly increased in mice injected with hPSC-LECs (H9 and BJ1) compared to the hLEC-control. ***p<0.001. Illustration in panel A was adapted with permission from [178]. (**b**) Fibrin/Collagen I hydrogels were used to generate dermo-epidermal skin grafts with blood and lymphatic capillaries. After 14 days post-transplantation, anastomosis occurred either as a “direct connection” (arrows) or as a “wrapping connection” (arrowheads). Dashed lines indicate the dermo-epidermal junction. Human lymphatic vessel (human podoplanin stained in red), rat lymphatic vessel (rat podoplanin stained in green), and nucleus stained in blue. Scale bars are 50 μm. Illustration in panel B was adapted with permission from [50]

The exciting discovery of human induced pluripotent stem cells (hiPSCs) enable the derivation of patient-specific LECs for cell therapy, drug screening, and tissue engineering applications. Various protocols to derive hiPSCs into BECs [175–177] can be optimized to further differentiate BECs into LECs. LECs derived from hiPSCs have been shown to help in wound healing by inducing lymphangiogenesis and lymphvasculogenesis *in vivo* (Fig. 4a) [178]. These LECs were derived and isolated from hiPSCs using a mouse fibroblast (OP9)-assisted culture system utilizing the VEGF-A, VEGF-C, and EGF, followed by FACS-sorting using LYVE-1 and PDPN [178]. A summary of methods used to derive LECs is shown in Table 1. Most of the methods that differentiate LECs from hiPSCs have relied on an embryoid body (EB) intermediate, which entails spontaneous differentiation to a complex cell mass in suspension, which requires subsequent isolation of cell based on specific markers [178, 179]. Other methods incorporate co-culture with mouse fibroblasts, which is less controllable and not suitable for clinical application [172, 178]. Therefore, there is a greater need to generate clinically-relevant LECs using a xeno-free and well-defined culture condition for therapeutic lymphangiogenesis [175].

**Table 1:** Summary of Protocols to Differentiate LECs

Cell Types	Methods	Results	Ref.
Healthy patient fibroblast: breast & abdominal	Isolated transcriptomes from LECs and BECs using FACS and microarray technology	Established complete transcriptomes of isolated LECs, BECs, and other skin cell types Novel endothelial cell subtype-restricted functions are influenced by the tissue environment	[195]
E14g2a Embryonic Stem (ES) Cell	On OP9 stromal cells, VEGFR2^+^ cells from ES cells differentiated to LECs with expressing of prox1, VEGFR3, LYVE1, and podoplanin	Differentiation of LECs from ES cell	[172]
Human ES cells and human iPSCs	OP9 assisted cell culture with VEGFA, VEGFC, and EGF LECs isolated using LYVE-1 and PDPN in FACS-sorting	Generation of LECs from hiPSCs and hESCs lymphangiogenesis and lympvasculogenesis as a function of LECs *in vivo* enhanced wound healing	[198]
Murine R1 ES cells	Murine R1 ES cells cultured on mitotically inactivated primary mouse embryonic fibroblast Embryoid bodies (EB) were isolated from embryonic stem cells Embryoid bodies stained using antibodies for LYVE-1, CD31, MECA-32, and PROX-1	LECs expressing CD31, PROX-1, and LYVE-1 differentiated 18 days after embryoid body formation Lymphatic vessel formation using VEGFA and VEGFC	[196]
hPSC	Used a monolayer culture of hPSCs hPSCs differentiated to early vascular cells which then matured to early endothelial cells and pericytes	hPSCs induced to codifferentiate into early vascular cells Early vascular cells mature to endothelial cells and pericytes and organize themselves into microvascular networks in a pre-engineered matrix (HA hydrogels)	[195]

SMCs have an important role in human tissues. Their normal function is necessary for the basal function of many organs such as the intestine and vascular system [180]. However, it should be noted that the accumulation of SMCs also lead to disease phenotypes such as neointimal hyperplasia [181–183]. Previously, SMCs use in cellular therapeutics has been limited due to limitations of a reliable source of SMCs. As previously mentioned, adipose tissue contains many different cell types and is an important source of multipotent cells [180, 184]. Adipose-derived cells and hiPSCs can be used to derive SMCs that exhibit all the SMCs markers presently known [175, 180, 185, 186]. These SMCs differentiated cells can respond to pharmacologic agents through contraction and relaxation [180, 185]. Similar to adipose tissue, bone marrow has also been shown to contain tissue specific stem and progenitor cells [187]. These bone marrow derived cells contribute to wound healing and limb ischemia through neoangiogenesis [188, 189], lymphoid organ neovascularization [171], and vascularization during neonatal growth [190]. SMCs play an important role in the function of the collecting lymphatic system. SMCs are capable of both spontaneous and phasic contractions, functioning as a pump in the lymphatic system [191]. This allows the body to maintain fluid homeostasis through removal of interstitial fluid from the interstitial space [192, 193]. The function of SMCs in the collecting lymphatic system are regulated by the physical and chemical stimulus such as transmural pressure and sheer stress [55, 194].

## VIII. Techniques for Lymphatic Tissue Engineering

Large advances in therapeutic strategies that combine material engineering with biotechnological advances to promote vascular regeneration have occurred in recent decades [197–199]. While these vascular regenerative approaches may be applicable to lymphatic regeneration, special approaches for LV engineering must be developed due to the unique features and characteristics, such as unidirectional flow, differing microarchitecture, and specialized valves, of lymphatic tissue [5, 24].

Currently, most LV engineering and regenerative medicine efforts are focusing on methods that include cell-seeded scaffolds for vessel reconstruction, injecting stem cells, delivering pro-lymphangiogenic cytokines or chemokines to stimulate in vivo lymph vessel growth, or a combination of these techniques [24, 41]. The approaches for LV engineering include regenerating pre-existing LVs through promotion of lymphangiogenesis, ex vivo assembly of lymphatic grafts, and in situ assembly of lymphatic structures for in vivo development [143, 200]. Outlined below, and summarized in Table 2, are multiple approaches for LV engineering that have demonstrated potential.

**Table 2:** Summary of Approaches for Lymphatic Tissue Engineering

Technique	Method	Model system	Results	Ref.
Hydrogels	hLECS overlaid with Fibrin, Collagen I, and Fibrin-Collagen I composite hydrogels	In vitro	-In absence of fibroblasts, no capillary formation -When hLECs with 40% dermal fibroblasts, branching capillaries developed within 21 days in vitro	[58]
	Fibrin and collagen ratios varied in hydrogels	In vitro	-LECs organized the most extensively in fibrin-only hydrogels, with slender networks and narrow lumens -Fibrin hydrogels stable for only 6 days	[160]
	hLECs co-cultured with ASCs in fibrin hydrogels and supplemented with VEGF-C	In vitro	-In the presence of ASCs, LECs formed tubules and networks -25ng/mL VEGF-C supplementation improved network formation	[201]
	HA-hydrogel	Lewis rat	-Mice that received HA-hydrogel demonstrated decreased scarring and decreased collagen deposition -HA treated group's ejection fraction was rescued to almost pre-MI baseline	[202]
Biochemical Stimuli	LECs supplemented with VEGF-A and VEGF-C	In vitro	-In vitro formation of lymphatic capillaries -Increased density of lymphatic capillary branching, compared to factor-free medium	[50]
	VEGF-C administered with skin graft	Mouse	-Lymphatic regeneration temporally and spatially associated with pattern of VEGF-C they were exposed to	[43]
	VEGF-C administered with autologous lymph node transfer	Domestic pig (female)	-Induced lymphangiogenesis	[213]
	VEGF-C gene therapy	Mouse, Rabbit	-Regenerated damaged lymphatic networks -Reduced edema	[211, 214–218]
	ANGPT1/2/TIE2	Proposed	-Guide postnatal maturation of LVs	[222]
	TGF-β	Proposed	-Primary ligand in ALK1 pathway which regulates differentiation of premature LECs into mature LECs	[223]
	PDGF-B, HGF, and/or Adrenomedullin	Proposed	-Enhance proliferation, migration, and tubule formation of LECs	[222, 224, 225]
Co-culture	LECs seeded on sheets of fibroblasts	In vitro	-Stable 3D lymphatic capillary networks spontaneously organized without exogenous materials	[228]
	LECs and dermal fibroblasts co-cultured for six weeks	In vitro	-LECs spontaneously organized and formed vasculature that resembled native in vivo tissue -Microvasculature stable for multiple weeks	[226]
Interstitial Flow (IF)	IF through collagen gels containing phorbol 12-myristate 13-acetate	In vitro	-Induced blood and lymphatic endothelial cell organization	[232]
	Low level IF added to 3D fibrin matrix containing VEGF	In vitro	-Complex capillary morphogenesis -Computational model showed that IF created gradient of VEGF	[160, 235]
	IF applied to regenerating skin	Mouse	-Lymphatic vessels only formed in the direction of lymph flow	[236]
	Multichamber radial fluidic device that exposed LECs to IF	In vitro	-LECs formed multicellular, lumenized structures similar to natural lymphatic networks	[200]
Extracorporeal Shockwave Therapy (ESWT)	Ear lymphedema treated with low-energy shockwaves	Rabbit	-Increased expression of VEGF-C and VEGFR-3 -Decreased lymphedema	[239]
	Tail lymphedema treated with low-energy ESWT	Rat	-Increased expression of VEGF-C and bFGF -Decreased lymphedema	[240]
Scaffolds	Collagen and fibrin-based hydrogels vascularized with LECs in vitro, then implanted	Mouse	-Functional vessels developed 15 days after implantation	[220]
	Engineered fibrin-binding VEGF-C (FB-VEGF-C) that is slowly released upon demand of infiltrating cells	Mouse	-FB-VEGF-C act synergistically with IF to drive lymphatic capillary morphogenesis in vitro -Induce local lymphatic hyperplasia but do not remodel downstream collecting vessels	[244]
	Nanofibrillar collagen scaffolds placed across lymphedema area to guide lymphatic regeneration	Porcine	-Increased number of lymphatic collectors in the proximity of scaffold -Bioimpedance ratio improved, implying that functional lymphatic drainage was restored	[245]
Combinatorial	Combinations of gelatin hydrogels, VEGF-C supplementation, and ESWT used to treat lymphedema	Mouse	-Greatest lymphatic vessel formation and greatest decrease in lymphedema resulted when all three approaches (hydrogels, VEGF-C, and ESWT) were combined	[250]

### Hydrogels

Hydrogels are water-based biomaterials that can incorporate cells or growth factors to initiate vascular network formation for in vitro or in vivo applications [24] and have demonstrated success in vascular regeneration in vitro applications [199]. Hydrogels can be employed to generate functional lymphatic capillaries, and multiple approaches have reported LECs forming networks in 2D and 3D experiments.

When a monolayer of human LECs (hLECs) were cultured and then overlaid with collagen type I or fibrin hydrogels, lymphatic capillaries formed within 21 days in vitro [50]. Fibroblasts were required in this model, as capillary formation in the absence of fibroblasts did not occur, but branching capillaries developed when hLECs were cultured with 40% human dermal fibroblasts [50]. In another experiment where hydrogels of varying ratios of fibrin and collagen were created, the importance of matrix selection with regards to the specific tissue engineering application was highlighted. While BECs organized the best in compliant collagen-containing hydrogels, LECs organized the most extensively in fibrin-only hydrogels [160]. In addition to different matrix preferences of BECs and LECs, different architectures have been observed between these two endothelial cell populations. While BECs formed thick, branched networks with wide lumens, LECs formed slender, overlapping networks with narrow lumens [160]. These differences between BECs and LECS highlight how techniques from vascular engineering can be used as a starting platform for lymphatic engineering but must be adapted and optimized.

**Fig. 5:**
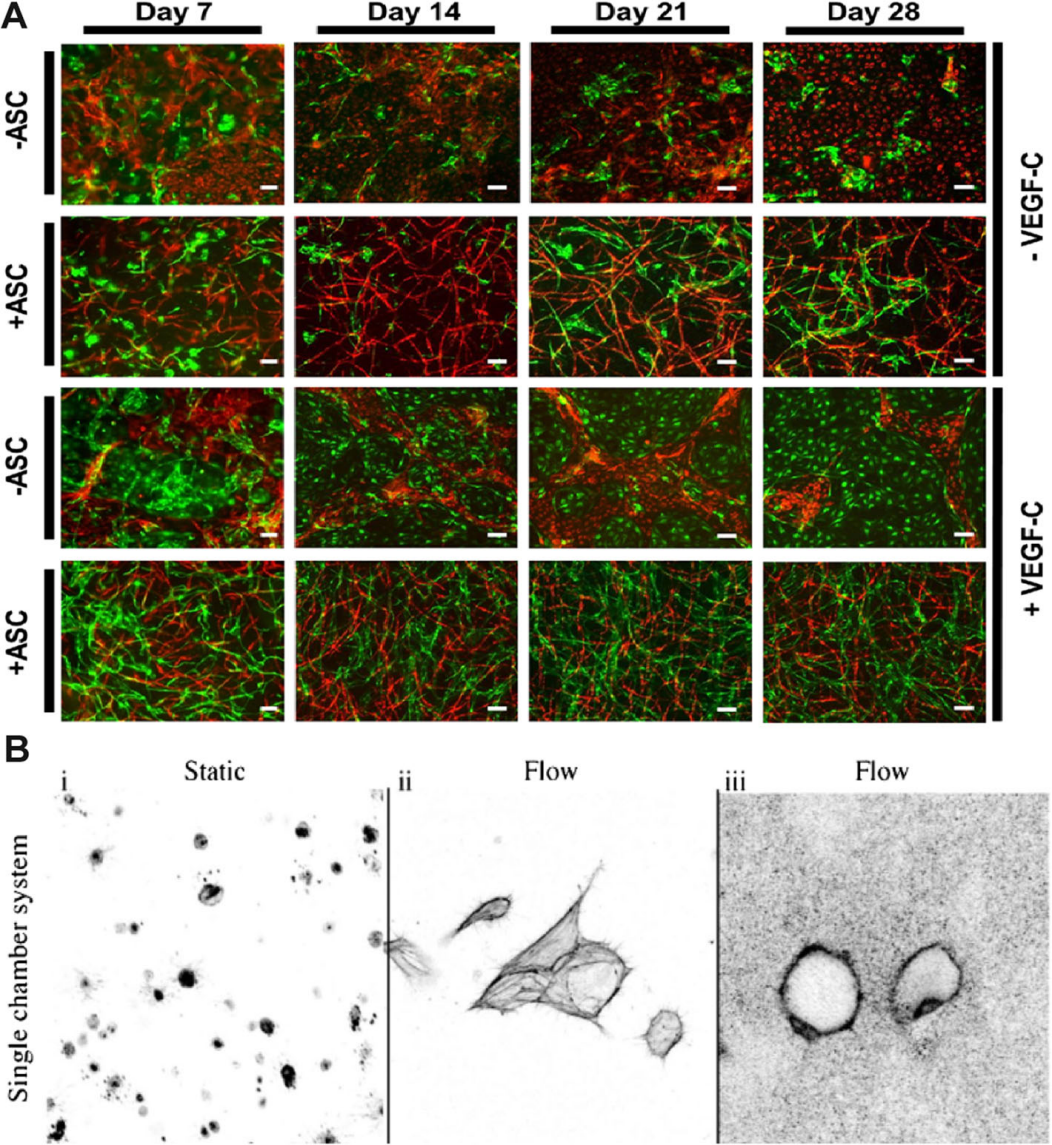
(**a**) In the presence of ASCs and a fibrin hydrogel system, LECs (green) and BECs (red) form networks that are distinct from each other. With the supplementation of VEGF-C, LECs form denser networks. Scale bars are 100μm. Illustration in panel A was adapted with permission from [201]. (**b**) With the addition of interstitial flow to the culture chamber, LECs formed capillaries after five days of continuous flow. Confocal imaging shows the multicellular networks(ii) and confocal reflectance indicates the networks contain lumens (iii). Illustration in panel B was adapted with permission from [231]

Beyond using fibrin and collagen I hydrogels for in vitro studies on LEC morphogenesis, hydrogels can also be used to generate bioengineered dermo-epidermal skin grafts with blood and lymphatic capillaries. When these engineered skin grafts were transplanted to a nude rat, the engineered human lymphatic capillaries anastomosed to the rat's lymphatic plexus and supported fluid drainage, suggesting that these skin grafts could be applied to patients suffering from severe skin defects [50] (Fig. 4b**)**. Moreover, hLECs can also be co-cultured with adipose-derived stromal cells (ASCs) to generate 3D networks. The need for cell-cell contact between hLECs and ASCs was highlighted as networks did not form in the absence of ASCs. hLEC and ASC co-cultures were additionally supplemented with VEGF-C to promote network formation. Additionally, a tri-culture system was used in these fibrin hydrogels, and after 28 days, distinct LEC and BEC networks formed in the presence and supplementation of ASCs and VEGF-C (Fig. 5a) [201].

Hyaluronic acid-based hydrogels (HA-hydrogels) have particularly shown great promise, either as a stand-alone therapy or as a scaffold to deliver molecules and cells [202]. HA is a non-sulphated glycosaminoglycan that contains repeating disaccharide units of N-acetylglucosamine and glucuronic acid [203]. HA is ubiquitous in the ECM, non-immunogenic, exists in a wide range of molecular weights from 100-800,000kDA [204, 205], and has become an important component in biomaterials for cellular therapy and tissue engineering [206–209]. HA-hydrogels demonstrate regenerative potential and can be employed as a cardiovascular therapy [210]. In a MI model in Lewis rats, MI was induced and HA-hydrogels were subsequently injected into the peri-infarct region. Compared to the control group, mice that received HA-hydrogels demonstrated decreased scarring and a decrease in collagen deposition, as well as an 18.2% increase in the ejection fraction which returned it close to the pre-MI baseline ejection fraction [202]. Since, LECs predominantly express LYVE-1, the unique binding receptor for HA, using HA-based hydrogels for therapeutic lymphangiogenesis could be an attractive strategy.

### Biochemical Stimuli

Due to VEGFR3’s role in lymphangiogenesis, the VEGF-C/VEGFR-3 axis is widely proposed as a high potential target to promote lymphatic capillary formation [24]. Transient overexpression of VEGF-C has been observed to increase growth, differentiation, and maturation of LECs, creating functional LVs with valves and SMC coverage [211, 212]. Bioactiving scaffolds with lymphangiogenic specific cues could aid lymphatic growth and also improve outcomes in both congenital and acquired lymphedema [24].

When LECs were supplemented with VEGF-A and VEGF-C, formation of lymphatic capillaries in vitro was observed, as well as increased density of lymphatic capillary branching, compared to factor-free culture medium [50]. When mice received skin grafts for LV generation, lymphatic regeneration was temporally and spatially associated with the patterns of VEGF-C expression that they were exposed to [43]. In another animal study on secondary lymphedema, VEGF-C treatment in combination with autologous lymph node transfers induced lymphangiogenesis [213]. VEGF-C gene therapy has also been shown to regenerate damaged lymphatic networks in situ and reduce edema [211, 214–218].

Overexpression of VEGF-C is a highly attractive therapeutic option, but supplementation levels must remain within physiological parameters as concentrations of VEGF-C well beyond physiological levels induce lymphatic hyperplasia and inhibit and increase in LV density [219, 220]. While VEGF-C overexpression induces lymphangiogenesis in regenerating tissues [221], VEGF-C alone is insufficient under physiological conditions for increasing long-term lymphangiogenesis [222]. Despite the promise of VEGF-C supplementation, therapies solely based on VEGF-C will not be successful for treating secondary lymphedema because additional mediators are required in order to stabilize the lymphatic vasculature [24].

Other biochemical targets for promoting lymphangiogenesis include ANGPT1/2/TIE2 signaling which could guide postnatal maturation of LVs [222], the ALK1 pathway which regulates the differentiation of premature LECs into mature LECs [223], TGF-β which is the primary ALK1 ligand [24], and adrenomedullin [224], PDGF-B [222], or HGF [225] which are known to enhance proliferation, migration, and tubule formation of LECs. It has also been proposed that a combination of these factors and VEGF-C could be used in a timed-release strategy where VEGF-C would provide the initial cues and then additional molecules would provide an extended time of cues [24]. Some molecules, such as PDGF-B, enhance both angiogenesis and lymphangiogenesis while others, such as CCBE1, can stimulate only lymphangiogenesis without impacting angiogenesis [226]. If trying to engineer larger LVs, EphB4 and EPHRIN receptor could be investigated as they have been shown to regulate lymphatic development and could positively impact valve formation [227].

### Co-culture

When LECs were seeded onto feeder sheets of fibroblasts, stable 3D lymphatic capillary networks spontaneously organized without the addition of any exogenous biomaterials or growth factors. This method highlights how fibroblast-derived VEGF-C and HGF induced LEC proliferation and tube formation [228]. Another method for the formation of stable 3D lymphatic capillary networks without any exogenous materials or growth factors involves coculturing human LECs with dermal fibroblasts in a five stage protocol that requires six weeks. From this method, LECs spontaneously organized and formed vasculature that exhibited the major structural and cellular features of native in vivo human dermal lymphatic microvasculature. While this technique requires six weeks for the lymphatic vasculature generation, the resulting microvasculature has been observed to remain stable for many weeks [229].

### Interstitial Flow

The lymphatic system is incessantly exposed to and stimulated by fluid flow and pressure due to its role in clearing interstitial fluid [5]. Due to this role, it has been hypothesized that interstitial flow may regulate lymphatic capillary regeneration [4]. In 2003, a circumferential dermal regeneration model in the tail of a mouse was used as the seminal study on the role of interstitial flow in lymphangiogenesis [230]. Interstitial flow is highly heterogeneous in nature and results from Starling forces between the capillary, interstitial, and lymphatic compartments [4]. Capillary morphogenesis, fibroblast remodeling of the extracellular matrix (ECM), and tumor cell migration are affected by interstitial flow [231]. It has been suggested that the loose cell-cell junctions in native lymphatic capillaries may intrinsically result from interstitial flow [232]. In the absence of lymph flow through a regenerating region, LVs will fail to organize [233].

Interstitial flow has been identified as a stimulator of lymphatic capillary morphogenesis [232, 234]. Previously, interstitial flow through collagen gels containing phorbol 12-myristate 13-acetate was shown to induce both blood and lymphatic endothelial cell organization [232]. When low level interstitial flow was added to a 3D system, comprised of VEGF covalently bound to a fibrin matrix, complex capillary morphogenesis resulted from the synergization between interstitial flow and VEGF [160]. Computational models of VEGF release from this fibrin matrix suggest that interstitial flow creates directional transcellular protein gradients, aided by diffusion and convection, that endothelial cells directionally sense and respond to [235]. In a model of regenerating skin, epidermal regeneration and angiogenesis occurred on both ends of the regenerating tissue, while lymphatic vessels only formed in the direction of the lymph flow [236].

Beyond alignment of LECs, interstitial flow also increased fibroblast alignment [4]. Examining natural in vivo functions, increased interstitial flow and fibroblast alignment are observed in tissue remodeling and wound healing [237]. Interstitial flow may also dictate cellular preferences for specific scaffolds or substrates. Fibrin-only matrices had the lowest hydraulic permeability when compared to collagen-only and fibrin-collagen-composite matrices, and fostered the greatest LEC organization. Additionally, greater capillary morphogenesis was observed in more compliant matrices, independent of soluble protease or VEGF concentrations, suggesting that differences in organizational behavior can be due to the resistance to fluid flow through the matrix [160].

In a multichamber radial fluidic device that exposed LECs to interstitial flow, LECs formed multicellular, lumenized structures that represented natural lymphatic networks (Fig. 5b). This fluidic chamber allowed for live imaging, multiple experiments to be performed simultaneously, and long-term cell culture. The addition of VEGF could also further increase the vessel density [231].

Given the demonstrated effect of interstitial flow on lymphatic morphogenesis, it could be debated that interstitial flow should be a design principle for in vivo capillary engineering [4]. With the aid of microfluidics to incorporate interstitial flow into a 3D LEC culture system, a more representative model can be designed in order to mimic the native environment and account for the multiple stimulatory factors of LEC morphogenesis.

### Extracorporeal Shockwave Therapy

Originally used to remove kidney stones [238], extracorporeal shockwave therapy (ESWT) has recently been shown to aid the regeneration of LVs by increasing cell permeability and expression of growth factors such as VEGF-C [5]. In a rabbit model, dysfunctional LVs in the ear were treated with or without low-energy shockwaves, and those treated with shockwaves showed increased expression of VEGF-C and VEGFR-3, as well as decreased lymphedema [239]. Similarly, decreased lymphedema and increased expression of VEGF-C and bFGF was observed in the tails of rats that received low-energy ESWT [240].

### Scaffolds

**Fig. 6:**
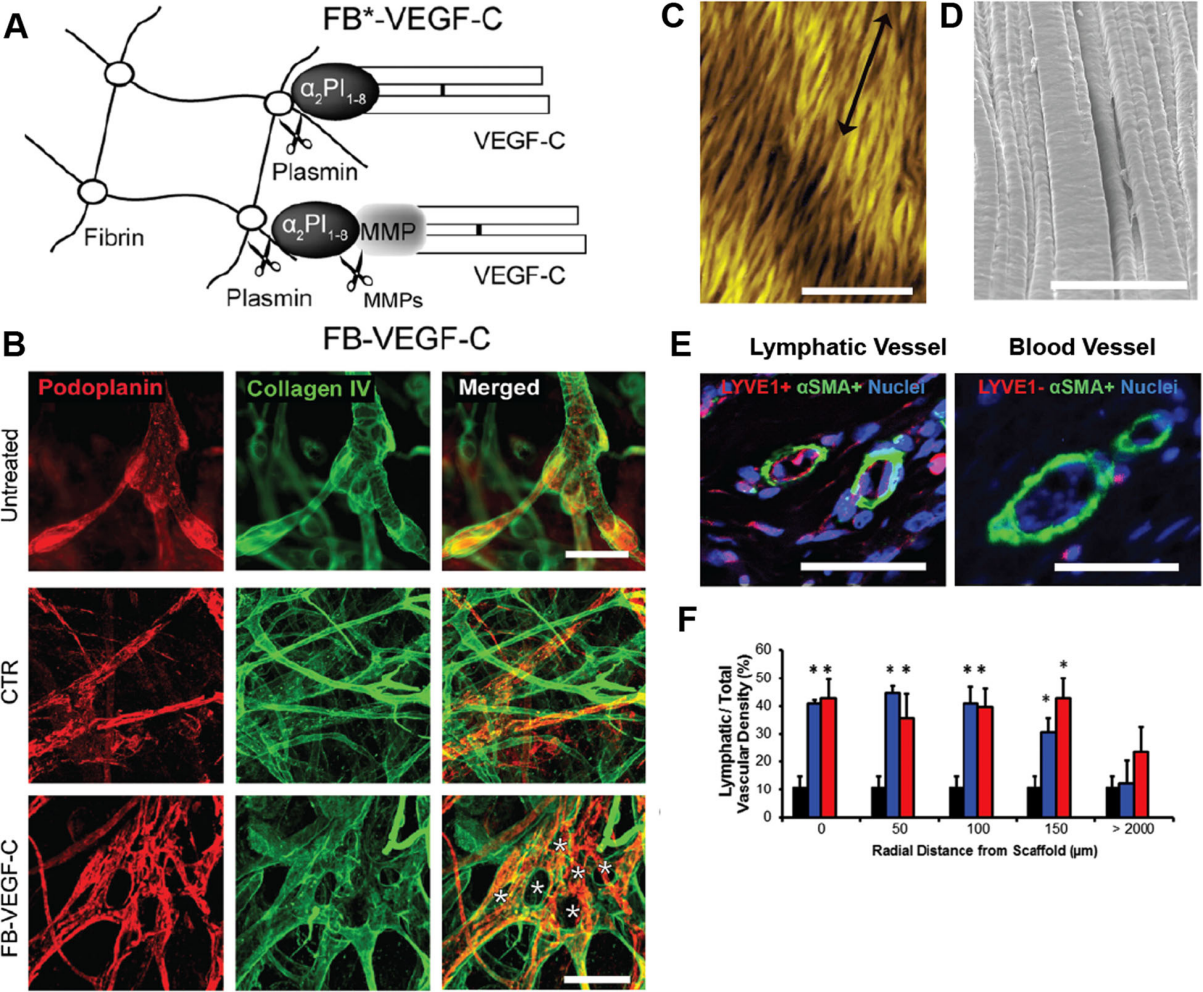
**.** (**a**) Engineered fibrin-binding variant of vascular endothelial growth factor C (FB-VEGF-C) that is slowly released upon demand by infiltrating cells. VEGF-C release is enabled by plasmin cleavage of fibrin or MMP cleavage of the additional MMP substrate peptide fused between the α_2_-PI_1-8_. (**b**) Confocal images of untreated dorsal ear dermis and 21 days after treatment with the fibrin gel (CTR) or FB-VEGF-C. Images show podoplanin (red), collagen IV (green) staining. Star indicates pillar formation on the FB-VEGF-C group. Scale bars are 50 μm. Illustration in panel A and B was adapted with permission from [244]. BioBridge, alligned nanofibrillar collagen scaffolds characterized using (**c**) atomic force microscopy (scale bar is 50 μm) and (**d**) scanning electron microscopy (scale bar is 20 μm). (**e**) At 3 months after implantation in a porcine model of acquired lymphedema, BioBridge and VEGF-C treated group show lymphatic and blood vasculatures. Scale bars are 50 μm. (**f**) Lymphatic fraction of total (blood + lymphatic) vascular density in percent (*n*>3), *p<0.05 versus untreated irradiated tissue (control group). Illustration in panel C-F was adapted with permission from [245]

In situ tissue engineering is a common technique in tissue engineering and utilizes a patient’s native circulating cells to infiltrate and degrade an implanted cell-free scaffold. Upon scaffold degradation, the remaining tissue can function just as the natural host tissue would [197, 241]. Scaffolds can be created from natural proteins or synthetic polymers and have been shown to maintain their lumen for up to 1 year after implantation [241]. Another cell-free scaffold approach uses scaffolds to guide and direct cellular behavior. Protein engineering can be used to generate highly angiogenic peptide nanofibers [242], VEGF-mimetic supramolecular nanostructures [243], and on-demand release of VEGF-C from fibrin scaffolds in the presence of plasmin or MMP [244]. Remarkably, implanted fibrin containing fibrin-binding (FB-VEGF-C), but not free VEGF-C, could stimulate local lymphangiogenesis in a dose-dependent manner (Fig. 6 a-b). On a different study, when nanofibrillar collagen scaffolds and VEGF-C were placed across an area affected by lymphedema, an increased number of lymphatic collectors were identified surrounding the scaffold three months after implantation (Figure 6 c-f). The bioimpedance ratio of the porcine subjects that received these collagen scaffolds was significantly improved, implying that functional lymphatic drainage in the treated area was restored [245].

Alternatively, cells from a patient could be isolated and then assembled ex vivo into a composite containing a scaffold with embedded, connected vascular and lymphatic capillaries which would then be implanted back into the patient [24]. This ex vivo approach has demonstrated success where previously, collagen and fibrin-based hydrogels were vascularized with lymphatic microvessels in vitro and then implanted in vivo where they became functional as early as 15 days postimplantation [220]. While LECs can organize into microvessels in both fibrin and collagen based matrices, they organize more extensity in fibrin-only-based matrices [160]. LECs can also attach to unwoven polyglycolic acid scaffolds [246]. In order to simulate lymph nodes, nonwoven polyamides, agarose matrix sheets, and macroporous cellulose microcarriers within an in vitro bioreactor have been utilized [247, 248].

### Combinatorial Techniques

In order to form functional tissue systems, biochemical, biomechanical, and cellular components need to be integrated [161], as it has been shown in many cases that biomechanical cues can act in synergy with biochemical cues and resultantly affect morphogenesis [160]. While VEGF-C is required for lymphatic capillary morphogenesis, interstitial flow is required for capillary organization and perfusion [220, 234, 249]. Alternatively, LECs can be embedded in a matrix and undergo increased proliferation with the addition of pro-lymphangiogenic growth factors, interstitial flow, or ESWT [5]. In a mouse model of lymphedema, the effects of differing combinations of gelatin hydrogels, VEGF-C supplementation, and ESWT were investigated. The greatest lymphatic vessel formation, decrease in lymphedema, and increase in VEGF-C and VEGFR-3 expression was observed when all three techniques were combined [250].

## IX. Verifying Lymphatic Phenotype and Functionality

In order to confirm a lymphatic phenotype, the presence of anchoring filaments and all major lymphatic markers should be verified. A discontinuous basement membrane, lack of mural cell coverage, and presence of anchoring filaments should also be examined, as they are identifying characteristics of lymphatic microvessels [3]. Beyond the phenotype, several parameters should be evaluated to confirm the functionality. The ability of the lymphatic structure to respond to both lymphangiogenic and anti-lymphangiogenic stimuli, take up fluid from the interstitial space, drain fluid, and respond to interstitial pressure variations should be evaluated [50].

To test the LV reaction to interstitial pressure fluctuations and maintain fluid homeostasis, Evans blue dye can be injected into the prevascularized scaffold and then CD31+ and LYVE-1+ lymphatic capillaries monitored for uptake of the dye from the extracellular space. The presence of anchoring filaments can also indicate the ability of the LVs to respond to interstitial pressure variations and fluid accumulation in vivo. Lymphatic drainage experiments have been performed in vivo by injecting Evans blue dye into grafts 15 days after transplantation and then analyzing the grafts 30 minutes after the dye injection. Upon analysis in these experiments, five times more dye was retained in hydrogels containing human lymphatic and blood capillaries, as compared to the fibroblast only hydrogel control, and indicated lymphatic drainage [50]. In addition to these functionality tests, accurate and robust methods to visualize LVs is a necessity. One method to detect and visualize LVs has been to use transgenic Prox1-Cre-tdTomato reporter mice [251]. The diameter of LVs can also be monitored, as an increased vessel diameter has been correlated with expansion of the lymphatic network [48].

### X. Specific Applications of Engineered LVs (summarized in Table 3)

***Cardiac Repair***

**Table 3:** A summary of therapeutic targets that could benefit from lymphatic tissue engineering

Application	Approach and targets	Clinical outcome	Ref.
Cardiac Repair	Induce lymphangiogenesis to create a pathway for inflammatory cell efflux and promote wound healing	-Rescue lymphatic transport ability -Mobilize macrophages -Reduce inflammation and edema	[54, 48, 253–255]
	Deliver VEGF-C to promote lymphangiogenesis	-Smaller ventricular end-systolic volume and improved ejection fraction -Accelerated cardiac lymphangiogenesis and limited collecting vessel remodeling -Decreased cardiac inflammation, fibrosis, and dysfunction	[29, 48]
	Inject HA-based hydrogels into peri-infarct region	-Ejection fraction improved to almost pre-MI baseline levels -Decreased scarring and decreased collagen deposition	[202]
Neurological Conditions	Deliver VEGF-C to rescue impaired meningeal LVs and improve waste drainage from CNS	-Improved drainage of macromolecules -Improved brain perfusion -Improved learning and memory	[256]
	Rescue meningeal LVs to decrease amyloid-β deposition in the meninges	-Potential to slow onset of Alzheimer's and other age-related cognitive declines	[268]
Improved Skin Grafts	Incorporate LVs into skin grafts to treat full-thickness skin defects	-Improved perfusion of oxygen and nutrients in dermal component -Rapid integration, proliferation, and differentiation of skin graft	[261]
Improved Wound Healing	Implant hydrogel scaffolds embedded with LECs	-Accelerated healing rate -Enhanced lymphatic ingrowth -Potential to treat diabetic wounds	[260, 5]
Diabetes	Inhibit lymphatic-specific epsin expression	-Prevent degradation of VEGFR3 and negate diabetes-triggered downregulation of lymphangiogenesis	[266]

Following MI, there is a significant lymphangiogenic response which could be a therapeutic target to promote cardiac repair following MI and treat other cardiovascular diseases [29, 48]. Inducing lymphangiogenesis presents a novel method to treat the injured adult heart by providing a pathway for inflammatory cell efflux and to promote wound healing. When ischemic injury was experimentally simulated, cardiac lymphangiogenesis was observed [48]. Despite MI organically inducing intramyocardial capillary lymphangiogenesis, adverse remodeling occurred in collecting vessels and led to reduced cardiac lymphatic transport ability. As a result, both infarct and noninfarcted myocardium experienced edema for several months following MI [29].

A robust immune reaction that resembles the sequence in inflammatory functions and wound healing is associated with myocardial injuries [252]. In inflammatory settings, lymphangiogenesis is responsible for mobilizing macrophages and resolving tissue edema [54, 253]. In previous mouse models, reduced inflammation occurred following delivery of VEGF-C [254, 255].

When VEGF-C was administered following MI, improved cardiac function was observed. Following MI, wild-type and *Vegfr3*^*lacZ/+*^ reporter mice received recombinant VEGF-C, C156S, at days 0, 2, 3, 4, and 6. The lymphangiogenic response, quantified by the presence of X-gal, VEGFR-3, and Prox1, was measured at day 7 post-MI and a stronger response was observed in the VEGF-C treated samples, compared to the vehicle-treated samples. Longitudinal MRI was used to measure cardiac function, and smaller ventricular end-systolic volumes and improved ejection fraction were observed in the VEGF-C treated mice. These notable cardiac improvements were maintained for a minimum of 28 days following MI [48]. In another mouse study where albumin-alginate microparticles were used to deliver VEGF-C_C152S_ to the intramyocardial space, accelerated cardiac lymphangiogenesis and limited collecting vessel remodeling was observed post-MI. These responses occurred in a dose-dependent manner. Due to administration of VEGF-C_C152S_, cardiac inflammation, fibrosis, and dysfunction diminished and myocardial fluid balance improved [29]. In agreement with other disease models [211], these results demonstrate that growth-factor-induced cardiac lymphangiogenesis could improve the prognosis for an adult diseased heart [29, 48].

Post-MI therapeutic options are not solely limited to delivery of VEGF-C. After MI was induced in Lewis rats, HA-based hydrogels were injected into the peri-infarct region and returned the ejection fraction to almost pre-MI baseline levels. Using transthoracic echocardiography to evaluate cardiac function, an 18.2% (*P*<0.01) improvement in ejection fraction of gel-treated subjects, compared to control subjects, was measured [202]. Beyond improved ejection fractions, decreased scarring and decreased collagen deposition were observed in the gel-treated subjects. HA presents regenerative potential to be used independently or as a scaffold to deliver additional molecules or cells for the treatment of heart disease [202].

### Alzheimer’s Disease

Unique from other tissues, the parenchyma of the CNS does not have lymphatic vasculature and uses a paravascular route to remove waste products. Recent rediscovery and characterization of meningeal LVs has created interest in how waste is cleared from the CNS. In a mouse model, macromolecules from the CNS drained into the cervical lymph via meningeal LVs. When these meningeal LVs were impaired, both paravascular influx of macromolecules into the brain and efflux of macromolecules from the interstitial fluid was slowed down, resulting in cognitive impairment [256].

In an aged mouse model, delivery of VEGF-C improved meningeal lymphatic drainage of macromolecules from cerebrospinal fluid. This improvement in drainage resulted in improved brain perfusion, as well as improved learning and memory. In a transgenic mouse model of Alzheimer’s disease, disruption of meningeal LVs promoted amyloid- deposition in the meninges and exacerbated parenchymal amyloid- accumulation, suggesting that Alzheimer’s disease pathology and other age-related cognitive declines could be impacted or accelerated by meningeal lymphatic dysfunction. The results from these mouse models suggest that augmentation of meningeal lymphatic function could be a therapeutic target to prevent or delay age-related neurological diseases [256].

### Modeling Cancer Metastasis

In addition to cancerous cells, primary tumors also contain numerous stromal cell types [257], including endothelial cells which have been implicated in tumor promotion. Macrophages are recruited to the primary tumor microenvironment and increase tumor cell migration, invasion, and intravasation, which consequently increases the metastatic potential. Primary tumors also experience increased angiogenesis which creates more routes for metastatic cell escape. Breast cancer in particular has a high propensity to spread to the lungs, lymph nodes, and bone, and the lymph nodes may provide a fostering environment for cancer cells where they can acquire additional mutations and develop a higher metastatic potential [258].

The process of cancer cell invasion into the bloodstream is widely researched as it provides a route to the entire body for metastasis. Differing from blood vessels, the process of cancer cell invasion into the lymphatic system is considered a passive mechanism since there are no inter-endothelial cell tight junctions or an intact basement membrane that the cells must cross [259].

In addition to recruiting macrophages, primary tumors recruit mesenchymal stem cells (MSCs) which have the ability to differentiate into multiple cell types and enhance metastasis. It is currently accepted that MSCs contribute to cancer progression by promoting angiogenesis, as well as other mechanisms, but the role of MSCs and the lymphatic system in cancer progression is poorly understood [258]. If a functional tumor microenvironment model that incorporates LVs is created, the model can be probed to further our understanding of how the lymphatic system contributes to cancer metastasis and elucidate pathways that would be good candidates to target for treatment.

### Skin Grafts

In order to create a physiologically accurate skin graft and facilitate quicker skin regeneration post-transplantation, both blood and lymphatic vessels should be incorporated into skin grafts in order to reconstitute a full-thickness skin defect. Both immune cell recruitment and induction of lymphangiogenesis have been shown to accelerate skin regeneration [260]. By incorporating a network of capillaries into a skin graft, perfusion of the dermal component is improved and allows for rapid and efficient access to oxygen and nutrients. This increased perfusion results in rapid integration, proliferation, and differentiation of the skin graft [261].

Two populations of LECs were examined for their potential to form LVs and be incorporated into skin grafts; a pure population of human LECs and human dermal microvascular endothelial cells that contained a fraction of LECs. Both of these populations successfully developed lumen-forming lymphatic capillaries in vitro within 21 days when they were implanted in either fibrin or collagen type I hydrogels. Subsequently, these capillaries maintained their lumen and incomplete basement membrane when implanted in vivo. When grafted to the wounded back of nu/nu rats, these lymphatic capillary containing hydrogels anastomosed with the rat’s LVs within 14 days after transplantation. Additionally, the engineered lymphatic microvessels exhibited fibrillin^+^ anchoring filaments, which are necessary in order to respond to interstitial pressure changes, and supported fluid drainage, suggesting that these skin grafts could be used for patients with severe skin defects.

### Wound Healing

The wound healing process involves keratinocytes, fibroblasts, endothelial cells, macrophages, and platelets [27], and is impacted by lymphangiogenesis. When the removal of inflammatory cells and local debris is delayed, the wound healing process is impeded [50]. One method to overcome this impaired wound healing or to enhance lymphatic ingrowth following surgery, would be to implant hydrogel scaffolds that are embedded with LECs [5]. During wound healing, VEGF-C is upregulated [27] and highlights the potential of using VEGF-C to induce lymphangiogenesis and stimulate the wound healing process.

In a genetically diabetic mouse model, VEGF-C was administered via an adenoviral vector and an accelerated healing rate was observed in the VEGF-C treated mice. Diabetic foot ulcers are partially caused by impaired angiogenesis, and the improved healing rate in these diabetic mice demonstrates the therapeutic potential to use VEGF-C to treat diabetic wounds [260].

### Diabetes

In another diabetic mouse model, LECs isolated from diabetic wild-type mice demonstrated impaired proliferation, migration, and tube formation when treated with VEGF-C, compared to LECs isolated from diabetic LEC-iDKO mice. Increased LV growth in the corneas and subcutaneous Matrigel plugs was observed in diabetic LEC-iDKO mice, compared to the diabetic wild-type mice, following VEGF-C administration. Additionally, enhanced lymphangiogenesis was observed in LEC-iDKO mice, a variant that is deficient in epsins 1 and 2 on LECs [166].

In the presence of lymphatic-specific epsin loss, lymphangiogenesis is downregulated and increased tail edemas were observed in diabetic mice. Reactive oxygen species caused increased epsin expression. When epsin bound to VEGFR3 in the Golgi compartment, degrdation of VEGFR3 was promoted and caused the availability of VEGFR3 at the cell surface to be reduced [166]. This LEC-iDKO mouse model suggests that inhibited epsin expression prevents VEGFR3 from degradation and would negate diabetes-triggered downregulation of lymphangiogenesis. Targeting this pathway could be a novel therapeutic strategy for diabetes related complications [166].

## XI. Challenges of Engineering LVs

A perennial challenge for the field of tissue engineering is the vascularization of tissues and in vivo endothelial cell organization in order to form capillaries [5, 160]. One of the challenges associated with this goal of controlling in vitro or in vivo morphogenesis of cellular structures includes the need to accurately replicate the morphology and cellular organization of lymphatic vessels [5]. The complex architecture of LVs must be considered when designing LV engineering techniques. Advances in blood vessel engineering have been made, but the unidirectional flow, special valves, and differing structure of LVs require special approaches for LV engineering in addition to the generalized techniques that have been developed for creating blood vessels [5].

One universal approach for engineering LVs will be insufficient for multiple reasons. The anatomy and function of specific LVs varies, based on the vessel’s location in the hierarchy, and will require different approaches in order to accommodate the different structures and cellular organization. Additionally there are many pathologies that result in lymphedema, both primary, secondary, and patient-specific treatment strategies may be required or even multiple techniques for a single patient [24]. For example, VEGF-C has widely been studied as a method to stimulate lymphangiogenesis [262]. However, VEGF-C therapy alone would be insufficient to treat secondary lymphedema as additional mediators would be required to stabilize the lymphatic vasculature [24]. Beyond the need for additional mediators, the effect of VEGF-C inducing lymphangiogenesis has been shown to be transient and insufficient for long-term applications under physiological conditions [222], raising the need for multiple approaches in order to sustain a long-term solution. The underlying pathology and cause of lymphatic dysfunction, as well as the in situ disease microenvironment, may control the outcome of lymphatic regenerative medicine approaches [24].

It is hypothesized that without the incorporation of SMCs and pericytes, overall lymphatic vascularization or effective lymphatic host replacement would fail due to the lack of functional collecting vessels. In order to effectively decrease swelling or edema, both lymphatic capillaries and collecting vessels need to be functional because edemtaous areas are typically very large and will require drainage through larger vessels than and not solely capillaries [24]. While small diameter vascular grafts risk thrombosis, coagulation and collapsing due to a low flow rate [143] are the primary risks for lymphatic grafts. These risks should be accounted for in the design of scaffold materials to be used for LV engineering.

Another challenge to engineering LVs is the prerequisite of a viable LEC source. Recently, iPSCs have been differentiated into the lymphatic lineage, alleviating some of the previous challenge [178]. Prior to iPSC differentiation into the lymphatic lineage, the only source of LECs was to isolate LECs from the dermis, intestine, and lymph nodes [263, 264]. For in situ cellurization, in vitro culturing may be required instead of simply implanting cells, due to an absence of a critical number of circulating cells and the inability to infiltrate a bare scaffold [24]. If SMCs are unable to be effectively recruited from the circulation, in vitro culturing may also be required in order for these pacemaker cells to be programmed and properly conduct rhythmic contractions for the propulsion of lymph fluid [265].

Beyond challenges related to the structural organization, autoimmunity may pose a formidable challenge. Lymphangiogenesis is speculated to contribute to immune rejection, as it has been observed in autoimmunity-related chronic inflammatory disorders [7, 55] and in transplant rejection [266]. The correlation between lymphangiogenesis and immunity needs to be further understood though, as contradictory results have been observed. While blocking lymphangiogenesis may reduce rejection rates [56, 267], transplant function and lymphangiogenesis were positively correlated in a 1-year follow-up study of renal transplant recipients [268].

Despite the advances in LV engineering currently made and the promise they demonstrate for clinical use, the challenge of discovering the optimal parameters for LV engineering remain for future studies [41].

## XII. Future Outlook of Engineered LVs

For effective lymphedema treatments, viable options should be less invasive than current options and the native architecture, function, and properties should be matched to the desired lymphatic structure to be replaced or repaired. The host location as well as the hierarchy of lymphatic vessels, i.e. lymphatic capillaries versus collecting vessels, will dictate these parameters to be matched. While several research groups have reported successful generation of lymphatic capillaries, successful regeneration of larger lymphatic vessels has not yet been achieved. Additionally, most research efforts to date have excluded pericytes, SMCs, or valves from lymphatic vessel design which will need to be addressed in future efforts for the successful design of collecting vessels [24].

Therapeutic lymphangiogenesis or engineered LVs have the potential to improve many areas of medical treatment. For cardiovascular diseases, therapeutic lymphangiogenesis may be a new approach for treating patients after a disease onset or to minimize detrimental effects of cardiovascular disease [29]. In the case of age-related neurological diseases, including Alzheimer’s, augmentation of meningeal lymphatic function is speculated to be a potential therapeutic target that could delay or even prevent the onset of this class of disease [256].

Ultimately within the past few decades, a deeper understanding of the lymphatic system has been developed, along with crucial cell-specific markers. The signaling factors and receptors necessary for differentiation of LECs from pluripotent cells have also been identified, although more research is needed to gain a better understanding of how the lymphatic system is formed. Furthermore, adipose tissue and bone marrow have been identified as sources of pluripotent cells from which LECs and SMCs can be derived using a well-defined and xenofree differentiation protocol. Then, this reliable human cell sources can be used within a biologically rational synthetic and controllable matrix environment for therapeutic lymphangiogenesis. Collectively, given the important roles of lymphatic vasculature in regulating many vital organs, therapeutic lymphangiogenesis has the potential to revolutionize the way we understand, manage, and treat various diseases.

## Abbreviations

ANG2: Angiopoietin 2
ASC: Adipose-derived stromal cells
BEC: Blood endothelial cell
BMI: Body mass index
BM-MSC: Bone-marrow mesenchymal stem cell
BMP-9: Bone morphologic protein-9
CLEC-2: C-type lectin-like receptor 2
CNS: Central nervous system
CSF: Cerebrospinal fluid
EB: Embryoid body
ES: Embryonic stem
ESWT: Extracorporeal shockwave therapy
HA: Hyaluronic Acid / Hyaluronan
HEVs: High endothelial venules
hLEC: Human lymphatic endothelial cells
hPSCs: Human pluripotent stem cells
IF: interstitial fluid
iPSC: Induced pluripotent stem cells
LECs: Lymphatic endothelial cells
LVs: lymphatic vessels
LYVE-1: Lymphatic vessel hyaluronan receptor-1
MAPCs: Multipotent adult progenitor cells
MI: myocardial infarction
MSC: Mesenchymal stem cells
muLECs: Meningeal mural lymphatic endothelial cells
PDPN: Podoplanin
PROX1: Prospero homeobox protein 1
VEGF-A: Vascular endothelial growth factor A
VEGF-C: Vascular endothelial growth factor C
VEGF-D: Vascular endothelial growth factor D
VEGFR-2: Vascular Endothelial growth factor receptor 2
VEGFR-3: Vascular endothelial growth factor receptor 3
